# Role of microRNA-199a-5p and discoidin domain receptor 1 in human hepatocellular carcinoma invasion

**DOI:** 10.1186/1476-4598-9-227

**Published:** 2010-08-27

**Authors:** Qingli Shen, Vito R Cicinnati, Xiaoyong Zhang, Speranta Iacob, Frank Weber, Georgios C Sotiropoulos, Arnold Radtke, Mengji Lu, Andreas Paul, Guido Gerken, Susanne Beckebaum

**Affiliations:** 1 Department of Gastroenterology and Hepatology, University Hospital Essen, 55 Hufeland Street, Essen, 45122, Germany; 2 Department of General, Visceral and Transplantation Surgery, University Hospital Essen, 55 Hufeland Street, Essen, 45122, Germany; 3 Institute of Virology, University Hospital Essen, 55 Hufeland Street, Essen, 45122, Germany; 4 Gastroenterology and Hepatology Center, Fundeni Clinical Institute, 258 Fundeni Street, Bucharest, 22338, Romania; 5 Department of Gynecology & Obstetrics, Guangzhou Women and Childrens Medical Center, Guangzhou, China

## Abstract

**Background:**

Micro-ribonucleic acid (miRNA)-199a-5p has been reported to be decreased in hepatocellular carcinoma (HCC) compared to normal tissue. Discoidin domain receptor-1 (DDR1) tyrosine kinase, involved in cell invasion-related signaling pathway, was predicted to be a potential target of miR-199a-5p by the use of miRNA target prediction algorithms. The aim of this study was to investigate the role of miR-199a-5p and DDR1 in HCC invasion.

**Methods:**

Mature miR-199a-5p and *DDR1 *expression were evaluated in tumor and adjacent non-tumor liver tissues from 23 patients with HCC undergoing liver resection and five hepatoma cell lines by the use of real-time quantitative RT-PCR (qRT-PCR) analysis. The effect of aberrant miR-199a-5p expression on cell invasion was assessed *in vitro *using HepG2 and SNU-182 hepatoma cell lines. Luciferase reporter assay was employed to validate *DDR1 *as a putative miR-199a-5p target gene. Regulation of *DDR1 *expression by miR-199a-5p was assessed by the use qRT-PCR and western blotting analysis.

**Results:**

A significant down-regulation of miR-199a-5p was observed in 65.2% of HCC tissues and in four of five cell lines. In contrast, *DDR1 *expression was significantly increased in 52.2% of HCC samples and in two of five cell lines. Increased *DDR1 *expression in HCC was associated with advanced tumor stage. *DDR1 *was shown to be a direct target of miR-199a-5p by luciferase reporter assay. Transfection of miR-199a-5p inhibited invasion of HepG2 but not SNU-182 hepatoma cells.

**Conclusions:**

Decreased expression of miR-199a-5p contributes to increased cell invasion by functional deregulation of *DDR1 *activity in HCC. However, the effect of miR-199a-5p on *DDR1 *varies among individuals and hepatoma cell lines. These findings may have significant translational relevance for development of new targeted therapies as well as prognostic prediction for patients with HCC.

## Introduction

Hepatocellular carcinoma (HCC) is the fifth most common malignancy worldwide and has an increasing incidence in western countries [1]. Although the risk factors for HCC are well characterized, the molecular pathogenesis of this particular tumor type is not well understood [2]. Micro-ribonucleic acids (miRNAs) represent an abundant class of endogenous small RNA molecules of 20-25 nucleotides in length [3] capable of mediating a vast gene regulatory network [4]. MiRNAs can regulate gene expression by direct cleavage of targeted messenger-RNAs (mRNAs) or by inhibiting translation through complementarity to targeted mRNAs at the 3'untranslated regions (UTRs) [5]. Computational analysis indicates that the total number of miRNAs may be greater than 1% of the protein coding genes in the human genome [6]. To date, 721 human miRNAs are annotated in the miRBase release 14.0 database [7]. Genes targeted by miRNAs control multiple biological processes in health and disease [8], including cancer development [9]. Accumulating evidence suggests that some miRNAs may function as oncogenes or tumor suppressors [10]. Recently, miRNA expression patterns have been investigated in HCC [11-16]. Although decreased expression of miR-199a-5p has been frequently demonstrated in HCC [11,12,15], functional analysis and translational relevance of this phenomenon has not been defined. The discoidin domain receptor (DDR) belongs to a novel class of receptor tyrosine kinases with a characteristic discoidin homology domain, stalk region, transmembrane region, juxtamembrane region, and kinase domain [17]. The DDR family consists of two members, DDR1 and DDR2, which can be alternatively spliced into five DDR1 isoforms (DDR1a-e) [18]. Over-expression of *DDR1 *was detected in several human cancers including breast [19], ovary [20], and lung [21]. The precise mechanism(s) by which these receptors may contribute to oncogenesis are not yet known. Targeted deletion of *DDR1 *in mice results in severe defects in placental implantation and mammary gland development [22], suggesting a potential role in cell migration and extracellular matrix degradation. Over-expression of *DDR1 *has been shown to increase the migration and invasion of hepatoma cells *in vitro *[23], implicating a causal role of DDR1 in promoting tumor progression. *DDR1 *is predicted to be a potential target of miR-199a-5p using publicly available PicTar (4-way), TargetScanS, and miRanda algorithms [24]. Thus, we postulated that aberrantly expressed miR-199a-5p may contribute to invasion by modulation of *DDR1 *expression in HCC patients.

## Methods

### Patients, tissues, cell lines, and cultures

HCC tissues and adjacent non-tumor tissues (NTs) used for qRT-PCR were collected from 23 HCC patients who underwent liver resection between December 2000 and March 2007 at the University Hospital Essen (Essen, Germany). Tissues were snap frozen in liquid nitrogen immediately after resection and then stored at -80°C until use. Demographic patient data are presented in Table 1. Tumor staging was performed according to the American Joint Committee on Cancer and International Union Against Cancer (AJCC/UICC) staging system (6th edition, 2002). Histological tumor grading was performed according to the Edmondson-Steiner classification: grade 1-2 (well differentiated), grade 3 (moderately differentiated), and grade 4 (poorly differentiated). The study was approved by the local ethics committee and all patients provided written informed consent. Hepatoma cell lines Hep3B (HB-8064), HepG2 (HB-8065), SK-HEP-1 (HTB-52) and SNU-182 (CRL-2235) were obtained from the American Type Culture Collection (Manassas, VA, USA) and cultured as recommended by the supplier. HuH-7 was a kind gift from Dr. Brigitte Pützer (Department of Vectorology and Experimental Gene Therapy, Biomedical Research Center, University of Rostock, Rostock, Germany). Primary human hepatocytes were obtained from ScienCell (San Diego, CA, USA).

**Table 1:** Demographic data of patients (n = 23)

Variable	Value ± SD^1 ^(%)
**Age **(years)	63.6 ± 15.5
	
**Gender **(male)	16 (69.5)
	
**Underlying liver disease**	16 (69.5)
Alcoholic liver disease	2 (8.7)
Cryptogenic cirrhosis	3 (13.1)
Hepatitis B	3 (13.1)
Hepatitis C	3 (13.1)
Nonalcoholic fatty liver disease	5 (21.6)
	
**Multiple tumoral nodules**	10 (43.5)
	
**Tumor size **- diameter of the biggest nodule (cm)	8.6 ± 4.3
	
**Tumor grade^2^**	
Well-differentiated (G1-2)	2 (8.7)
Moderately-differentiated (G3)	14 (60.9)
Poorly-differentiated (G4)	7 (30.4)
	
**UICC/AJCC^3^**	
I	3 (13.1)
II	6 (26.1)
III	13 (56.5)
IV	1 (4.3)

^1^Standard deviation

^2^Edmondson-Steiner

^3^International Union Against Cancer (UICC)/American Joint Committee on Cancer (AJCC) tumor-node-metastasis (TNM) staging system (6^th ^version, 2002)

### Generation of firefly luciferase constructs

Standard molecular biology techniques were used for generation of all constructs. For generation of a reporter vector bearing a human DDR1 fragment with putative miR-199a-5p binding sites, target sequences were cloned in the pMIR-REPORT Luciferase vector (Ambion). The pMIR-REPORT™ miRNA Expression Reporter Vector System consists of an experimental vector with a firefly luciferase reporter gene under the control of a cytomegalovirus promoter/termination system and an associated beta-galactosidase (β-gal) reporter gene control plasmid. The 3'UTR of the luciferase gene contains a multiple cloning site for insertion of predicted miRNA binding targets. By cloning a predicted miRNA target sequence into pMIR-REPORT, the luciferase reporter is subjected to regulation that mimics the miRNA target. The pMIR-REPORT β-gal reporter plasmid is used for transfection normalization. A human *DDR1 *3'UTR 457-bp fragment bearing all 4 putative binding sites for miR-199a-5p, which are identical among all the DDR1 splice variants, was generated by RT-PCR from total RNA extracted from HepG2 cells. Primers (*Spe*I and *Hind*III restriction sites are underlined) used to amplify this fragment were 5'-ACTAGTTTCCTTCCTAGAAGCCCCTGT-3' (forward primer) and 5'-AAGCTTCCCCAAT CCCAATATTTACTCC-3' (reverse primer) (Eurofins MWG Operon, Ebersberg, Germany). The PCR product was purified on agarose gel, isolated and first inserted into the pGEM-T easy vector (Promega) following the manufacturer's instructions. The pGEM-T plasmids were digested with the appropriate restriction enzymes and electrophoresed in agarose gel. The isolated insert was then excised from the gel, purified and subsequently subcloned in the *Hin*dIII/*Spe*I site of the pMIR-REPORT Luciferase vector. Plasmid constructs were verified for correctness by DNA sequencing using ABI PRISM^® ^3130 Genetic Analyzer (Applied Biosystems).

### Transfections

Pre-miR™ miRNA precursors of miR-199a-5p and non-targeting control miRNA precursors (Pre-miR™ miRNA Precursor Molecules-Negative Control #1) were purchased from Ambion, Inc. (Austin, TX, USA). Short interfering RNA (siRNA) against *DDR1 *mRNA (*DDR1*-siRNA) and a negative control siRNA were obtained from Qiagen (Hilden, Germany). Transfections of miRNA, siRNA as well as cotransfections of miRNA precursors and reporter vectors were performed using Lipofectamine 2000 (Invitrogen Corporation, Carlsbad, CA, USA). Conditions for HepG2 and SNU-182 cells were optimized to yield transfection efficiencies of 78% and 67%, respectively, with a cell viability > 80%. *GAPDH *knockdown and cell viability were both assessed by the KDalert™glyceraldehyde-3-phosphate dehydrogenase (GAPDH) assay kit (Ambion) for small RNA transfection.

### qRT-PCR

Total RNA from tissues and cells was prepared using the miRNeasy Mini Kit (Qiagen). The integrity and quantity of extracted RNA were assessed using Agilent RNA 6000 Nano Chip kit (Agilent Technologies, Waldbronn, Germany) with an Agilent 2100 Bioanalyzer (Agilent Technologies). Samples with RNA integrity number (RIN) values higher than 5 or higher than 8 were considered to have good and perfect total RNA quality, respectively, for downstream qRT-PCR application [25]. Therefore, RNA samples with RIN values lower than 5 were excluded from this study. Total RNA was treated with TURBO DNA-*free™*kit (Ambion) to remove any contaminating DNA from the RNA preparations. TaqMan^® ^qRT-PCR assays (Applied Biosystems, Darmstadt, Germany) were performed for qRT-PCR analysis to determine the quantity of mature miRNA. The primer and probe design for miRNA was done according to Chen et al. [26]. A multiplex reverse transcription reaction was carried out using 50 ng of total RNA and the TaqMan^® ^MicroRNA Reverse Transcription Kit as described by Tang et al. [27]. PCR was performed using TaqMan^® ^Universal PCR Master Mix without AmpErase^® ^uracil N-glycosylase according to the manufacturer's instructions. PCR efficiency of each set of primers and probes for each run was determined by a series of 2-fold cDNA dilutions of total RNA from primary human hepatocytes. *DDR1 *mRNA expression was determined by qRT-PCR assay using the SYBR^® ^Green QuantiTect RT-PCR Kit (Qiagen). Again, a series of 2-fold RNA dilutions was performed to determine PCR efficiency. All samples were run in triplicate on 96-well reaction plates with the iQ5 multicolor real-time PCR detection system (Bio-Rad, Hercules, CA, USA). Data were rescaled using qBase (version 1.3.5) [28], and the optimal reference genes were determined using *geNorm *(version 3.5) [29]. One sample was used as an inter-run calibration control to compare quantities between plates. Expression of mature miR-199a-5p was normalized with expression of small nucleolar RNA C/D box 44 (*RNU44*) and hydroxymethyl-bilane synthase (*HMBS*). Expression of *DDR1 *mRNA was normalized with optimal reference genes as previously described [30]. The sequences of primers and probes are shown in Table 2.

**Table 2:** Primers and probes for miR-199a-5p, *DDR1 *and reference genes.

Gene	Primer	Sequence (5'→3')
*miR-199a-5p*	RT	CTCAACTGGTGTCGTGGAGTCGGCAATTCAGTTGAGGAACAGGT
	Forward	ACACTCCAGCTGGGCCCAGTGTTCAGACTAC
	Reverse	CTCAACTGGTGTCGTGGAGTCGGCAA
	Probe	(6-FAM) TTCAGTTGAGGAACAGGT (MGB*)
*RNU44*	Forward	CCTGGATGATGATAGCAAATGC
	Reverse	GGCAATTCAGTTGAGAGTCAGTTAGA
	Probe	(6-FAM) ACTGAACATGAAGGTCTT (MGB*)
*HMBS* (TaqMan assay)	Forward	ATGTCTGGTAACGGCAATGC
	Reverse	GTACCCACGCGAATCACTCT
	Probe	(6-FAM) CTGCAACGGCGGAAGAAAACAGC
*DDR1*	Forward	AATCGCAGACTTTGGCATGAG
	Reverse	CGTGAACTTCCCCATGAGGAT
*GAPDH*	Forward	TGCACCACCAACTGCTTAGC
	Reverse	GGCATGGACTGTGGTCATGAG
*HMBS* (SYBR Green assay)	Forward	TGCAACGGCGGAAGAAAA
	Reverse	ACGAGGCTTTCAATGTTGCC
*HPRT1*	Forward	TGACACTGGCAAAACAATGCA
	Reverse	GGTCCTTTTCACCAGCAAGCT
*SDHA*	Forward	TGGGAACAAGAGGGCATCTG
	Reverse	CCACCACTGCATCAAATTCATG

*MGB: minor grove binder with non fluorescent quencher; *miR-199a-5p*: microRNA excised from the 5' arm of microRNA-199a precursor; *RNU44*: small nucleolar RNA, C/D box 44; *HMBS*: hydroxymethyl-bilane synthase; *DDR1*: discoidin domain receptor-1; *GAPDH*: glyceraldehyde-3-phosphate dehydrogenase; *HPRT1*: hypoxanthine phosphoribosyl-transferase 1; *SDHA*: succinate dehydrogenase complex, subunit A.

### Actinomycin D treatment for determination of mRNA stability

HepG2 cells were plated in 24-well plates and transfected with 10 nM of miR-199a-5p, miR-control or 100 nM si-*DDR1 *as a positive control. After transfection for 6 hours, cells were incubated with or without 4 μg/ml of actinomycin D (Sigma-Aldrich, Chemie GmbH, Steinheim, Germany) for additional 36 hours. Total RNA was extracted from cells and DDR1 mRNA was quantified by qRT-PCR described above. The expression levels of *DDR1 *are presented as values normalized against 10^6 ^copies of β-actin transcripts.

### Luciferase reporter assay

The pMIR-*DDR1*-3'-untranslated region (UTR) luciferase vector, containing the putative binding site for miR-199a-5p in the multiple cloning site within the 3' UTR of the luciferase gene in the pMIR-REPORT™ miRNA Expression Reporter Vector (Ambion) was constructed according to the manufacturer's instructions. HepG2 cells were plated at 2 × 10^5 ^cells/well in triplicate in 12-well plates. The pMIR-*DDR1*-3'-UTR construct (200 ng) together with β-gal expression vector pMIR-REPORT β-gal (200 ng) (Ambion) was cotransfected with Pre-miR™ miRNA Precursor Molecules or negative control miRNA precursors (Ambion). Luciferase assays and β-gal enzyme assays were performed 24 hours after transfection according to the manufacturer's protocol (Promega Corporation, Madison, WI, USA). Firefly luciferase activity was normalized to β-gal expression for each sample.

### Matrigel matrix invasion assay

Cell invasion assays were conducted using BD BioCoat Matrigel Invasion chambers (BD Biosciences Clontech, Heidelberg, Germany). Briefly, 48 hours after transfection 4 × 10^4 ^HepG2 cells or 2 × 10^4 ^SNU-182 cells were seeded into the top chamber with a Matrigel coated filter and 750 μl Dulbecco's Modified Eagle Medium containing 5% fetal bovine serum was used as a chemoattractant. Simultaneously, 100 μl of the cell suspension was seeded into 96-well plate in five replicates for cell number normalization using WST-1 assay. Inserts were incubated at 37°C with 5% CO_2 _for 22 hours. After incubation, cells that were still on the upper side of the filters were mechanically removed. Cells that migrated to the lower side were fixed with 100% methanol and stained with 1% toluidine blue (Sigma-Aldrich) in 1% borax (Sigma-Aldrich). Cells were counted in five fields for triplicate membranes at 10× magnification using an inverted optical microscope (Nikon ECLIPSE TS100, Nikon, Japan). The WST-1 assay was also performed and the invasion index was normalized to cell numbers.

### Cell proliferation assay

Cell proliferation was assessed using the water-soluble tetrazolium-1 (WST-1) assay following the manufacturer's protocol (Roche Applied Science). Cells were plated at the density of 8,000/well in 96-well plates (BD Biosciences, Rockville, MD), transfected as described above, and incubated at 37 °C. Cell proliferation was assessed after 72 hours in 5 replicates.

### Western blotting

Transfected cells were washed once with ice-cold PBS and lyzed in 1 × blue loading buffer (Cell Signaling Technology, Danvers, MA, USA) supplemented with a protease inhibitor cocktail (Roche Diagnostics). Protein samples were subjected to 10% of SDS-PAGE and blotted with primary antibody selectively recognizing DDR1 (C-20, Santa Cruz Biotechnology). To determine the amounts of loaded proteins, blots were probed with GAPDH (Cell Signaling Technology). Protein bands were visualized using ECL Plus Western blotting detection reagents (Amersham Biosciences, Buckinghamshire, UK) after incubation with appropriate HPR-conjugated secondary antibodies (Jackson Immuno Research Laboratories, West Grove, PA, USA), followed by exposure to Kodak Bio-Max films (Carestream Health, Paris, France).

### Statistical analysis

Data are expressed as mean ± SD unless otherwise indicated. Categorical data are described as frequency of the subjects with a specific characteristic. Chi-square test or Fisher's exact test was used for comparing categorical data and Student's t-test, Mann-Whitney-U-test, one-way ANOVA or Kruskal-Wallis test, when appropriate, was used for comparing continuous variables. Spearman's rank test correlation coefficient was used to measure the degree of association between two quantitative variables. Agreement of quantitative variables was evaluated by the correlation coefficient (r). To identify potential predictors of AJCC/UICC stage III-IV, univariate and multivariate analyses were performed. A forward stepwise selection method was used to select variables for the multivariate regression model. Two-tailed p-values less than 0.05 were considered statistically significant. Statistical analysis was performed using SPSS software version 12.0 (SPSS Inc., Chicago, IL, USA).

## Results

### Patient demographic data

A total of 23 patients were enrolled in the study and their characteristics are shown in Table 1. The mean age of the patients was 63.6 ± 15.5 years and 69.6% were male. There was no difference between cirrhotic and non-cirrhotic patients regarding number and size of tumors, AJCC/UICC stage, recurrence, or intrahepatic metastasis.

### Decreased miR-199a-5p expression in human HCC tissues and cell lines

**Figure 1:**
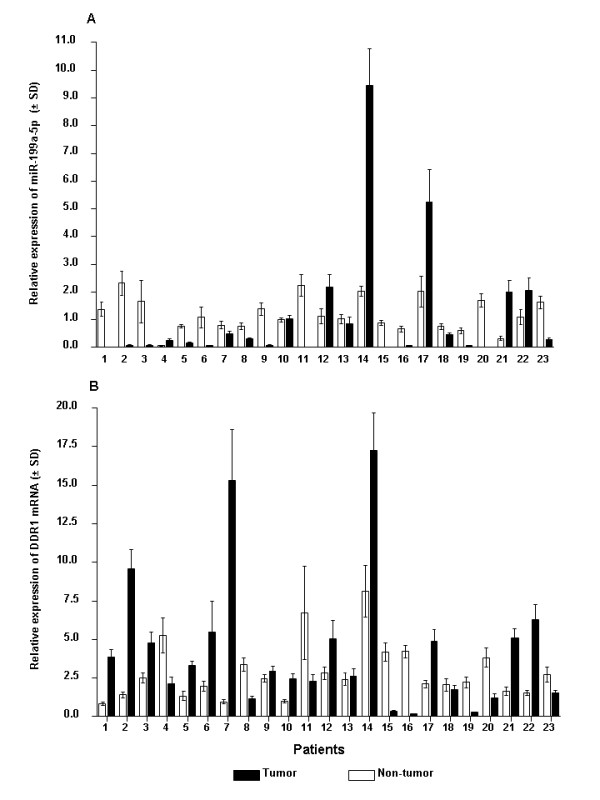
**Expression of miR-199a-5p and *DDR1 *in HCC specimens**. qRT-PCR analysis was performed using 23 paired surgical specimens of HCC tissues and adjacent normal tissues for miR-199a-5p **(A) **and *DDR1 ***(B)**. Expression levels normalized to expression of reference genes, *RNU44 *and *HMBS ***(A) **and *HMBS *and *GAPDH ***(B)**, are shown. Quantitative values representing the mean and SD from experiments performed in triplicate are presented.

**Figure 2:**
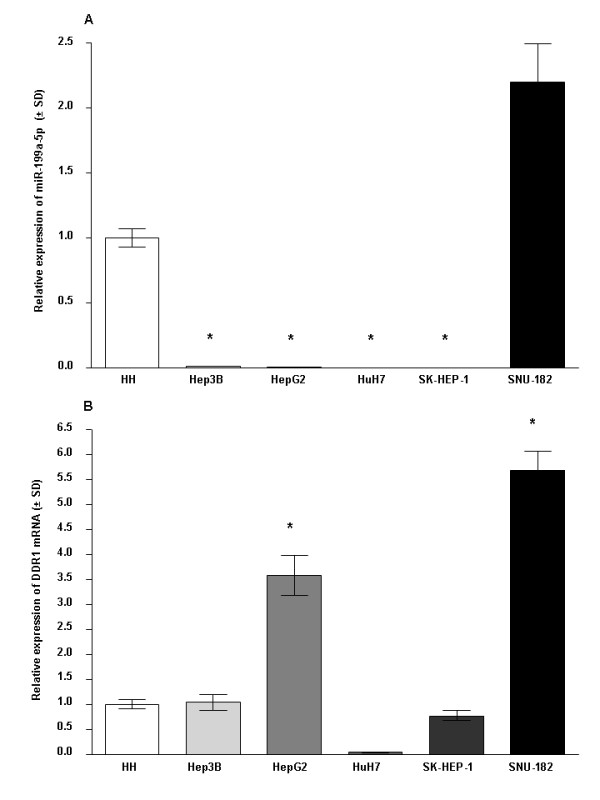
**Expression of miR-199a-5p and *DDR1 *in human HCC cell lines**. Expression levels of miR-199a-5p **(A) **and *DDR1 ***(B) **were determined by qRT-PCR analysis for 5 HCC cell lines (Hep3B, HepG2, HuH7, SK-Hep-1 and SNU-182) and primary human hepatocytes (HH). The expression levels were normalized to reference genes, *RNU44 *and *HMBS ***(A) **and *HMBS *and *GAPDH ***(B)**. Quantitative values representing the mean and SD from experiments performed in triplicate are presented. *p < 0.001 vs. primary human hepatocytes.

Matched tumoral and non-tumoral tissues of 12 patients were also included in another study, in which global miRNA profiling using miRNA microarray was performed (data not shown). MiR-199a-5p expression data acquired by real time RT-PCR correlated well with microarray data (r = 0.8077, p < 0.001), indicating that the qRT-PCR results are reliable. A significant down-regulation of miR-199a-5p (mean 0.15, SE 0.05, 95% CI 0.04-0.25, range 0.00-0.61) was noted in 15 of 23 (65.2%) HCC tissues compared to NTs (mean 1.24, SE 0.13, 95% CI 0.96-1.53, range 0.65-2.22, p < 0.0001, Figure 1A). No correlation between miR-199a-5p expression and clinical parameters was encountered. We next compared miR-199a-5p expression between hepatoma cell lines and primary human hepatocytes. Similarly to results obtained from HCC tissues, expression of miR-199a-5p was significantly (p < 0.001) decreased in four HCC cell lines (Hep3B, HepG2, HuH-7, and SK-HEP-1). However, increased expression of miR-199a-5p was found in another hepatoma cell line, SNU-182, compared with primary human hepatocytes (Figure 2A).

### Expression of *DDR1 *in human HCC tissues and cell lines

**Figure 3:**
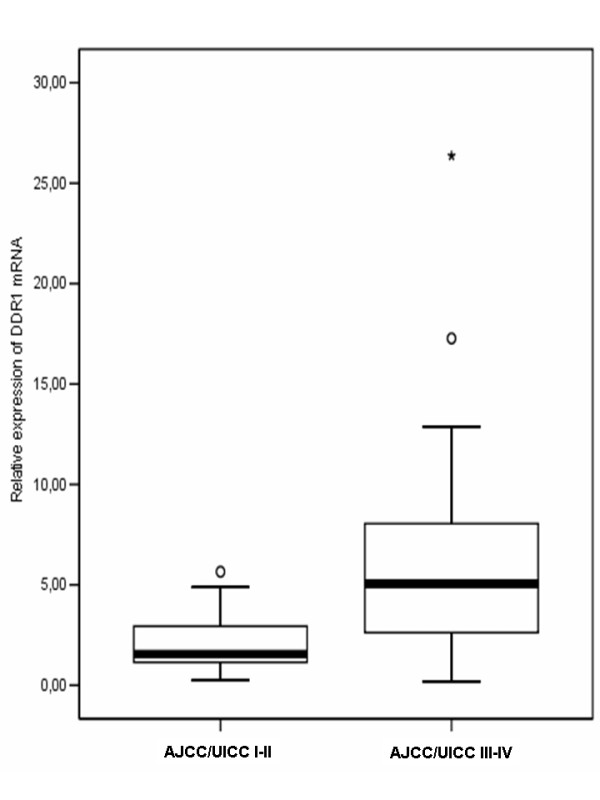
**Box plots of DDR1 expression in AJCC/UICC stages of HCC**. The relative expression levels of *DDR1 *in 23 human HCC tissues categorized according to AJCC/UICC stage I-II and AJCC/UICC stage III-IV are presented. The boxes enclose the interquartile ranges (IQRs), with the median values denoted by the horizontal lines. Circles (ο) represent outliers (values >1.5 × IQR). * p < 0.01 vs. AJCC/UICC stage I-II.

DDR1 was found to be significantly (p < 0.001) upregulated in HCC tissues (mean 8.49, SE 2.03, 95% CI 4.02-12.95, range 2.45-23.36) compared to matched NTs (mean 2.3, SE 0.58, 95% CI 1.02-3.57, range 0.74-8.15) in 12 of 23 (52.2%) patients (Figure 1B). Similarly, in two out of five hepatoma cell lines, HepG2 and SNU-182, *DDR1 *expression was significantly (p < 0.001) upregulated compared to primary human hepatocytes (Figure 2B). *DDR1 *expression was correlated significantly with both AJCC/UICC stage (r = 0.45, p = 0.03) and tumor status (r = 0.42, p = 0.04) in patients. Relative *DDR1 *expression values of AJCC/UICC stage III-IV patients (mean 7.11, SE 1.91, 95% CI 2.97-11.24, range 0.18-26.36) were significantly higher than those of AJCC/UICC stage I-II patients (mean 2.19, SE 0.64, 95% CI 0.71-3.67, range 0.25-5.65, p = 0.01) (Figure 3). Analyzing predictive factors for advanced tumor stage at diagnosis of HCC (Table 3), we found a significant association between AJCC/UICC stage III-IV and the following variables: histological grade of HCC (poorly differentiated) (p = 0.01), presence of macrovascular invasion (p = 0.04), and increased expression of *DDR1 *compared to NTs (p = 0.01). Multivariate logistic regression analysis performed for the variables that were significant (p < 0.05) in the univariate analysis, indicated that high *DDR1 *expression was the only single independent factor associated with HCC at AJCC/UICC stage III-IV (p = 0.04). There was no correlation between the expression of *DDR1 *and miR-199a-5p in tissues (r = 0.36, p = 0.09).

**Table 3:** Results of univariate analysis for factors associated with advanced HCC stage at diagnosis

Variable	AJCC/UICC stage I-II	AJCC/UICC stage III-IV	p value
Male gender	66.7%	71.4%	0.80
Age (years)	69.4 ± 8.0	60 ± 18.1	0.20
			
Multiple tumor nodules	44.4%	42.9%	0.94
Diameter of the biggest nodule (cm)	6.7 ± 4.4	9.8 ± 4.0	0.10
Viral infection	22.2%	28.6%	0.73
Cirrhosis	44.4%	28.6%	0.43
Poorly differentiated HCC (G4)	0%	50%	**0.01**
Macrovascular invasion	22.2%	64.3%	**0.04**
Mir-199a expression (median value)	0.07	0.36	0.17
DDR1 expression (median value)	1.54	5.05	**0.01**

### MiR-199a-5p differentially regulates the expression of DDR1 in HepG2 and SNU-182 cells

**Figure 4:**
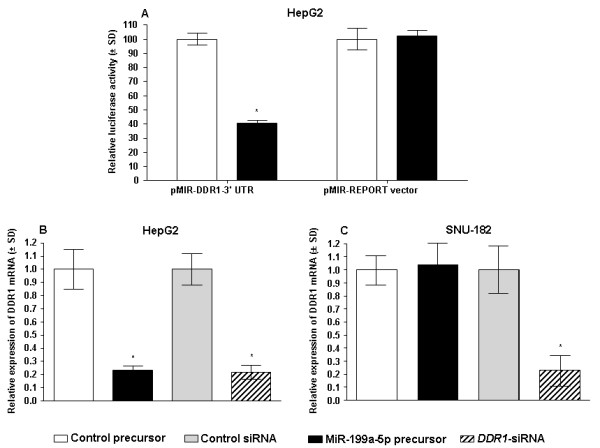
***DDR1 *is a target gene of miR-199a-5p**. **(A) **Cells were cotransfected with 200 ng of each vector (pMIR-*DDR1*-3'-UTR construct or empty pMIR-REPORT firefly luciferase reporter vector, together with beta-galactosidase (β-gal) expression construct pMIR-REPORT β-gal and miR-199a-5p precursor or control miRNA precursor using lipofectamine 2000. Luciferase assays and β-gal enzyme assays were performed 24 hours after transfection in triplicate. Firefly luciferase activity was normalized to β-gal expression for each sample. Data are shown as mean ± SD from 3 independent experiments. *p < 0.05 vs. respective control. **(B) **HepG2 or **(C) **SNU-182 cells were transfected with 10 nM miR-199a-5p precursor or 100 nM *DDR1*-siRNA. *DDR1 *mRNA expression was assessed by qRT-PCR and normalized to the expression of *HPRT1 *and *SDHA*. Cells transfected with control miRNA precursor or control siRNA were used as controls. Data are shown from 3 independent experiments each performed in triplicate as mean ± SD. *p < 0.05 vs. respective controls.

**Figure 5:**
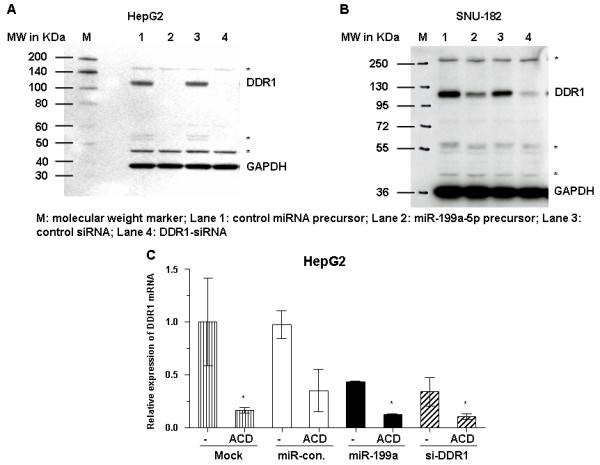
**MiR-199a-5p differentially regulates the expression of *DDR1***. **(A) **HepG2 or **(B) **SNU-182 cells were transfected with 10 nM miR-199a-5p precursor or 100 nM *DDR1*-siRNA. Cell lysates were obtained 48 hours after transfection for immunoblot analysis of DDR1 protein expression. GAPDH protein levels were determined as a loading control. Cells transfected with control miRNA precursor or control siRNA were used as controls. Lane M indicates the molecular weight marker and lane numbers 1 to 4 represent control miRNA precursor, miR-199a-5p precursor, control siRNA, and DDR1-siRNA respectively. Arrow, DDR1 protein; stars, protein bands resulting from nonspecific binding to DDR1-antibody. **(C) **For mRNA stability assays, HepG2 cells were plated in 24-well plates and transfected with 10 nM of miR-199a-5p, miR-control or 100 nM si-*DDR1 *as a positive control. After transfection for 6 hours, cells were incubated with or without 4 μg/ml of actinomycin D for additional 36 hours. Total RNA was extracted from cells and *DDR1 *mRNA was quantified by qRT-PCR described before. The expression levels of *DDR1 *are presented as values normalized against 10^6 ^copies of β-actin transcripts. Data are shown from 3 independent experiments each performed in triplicate as mean ± SD. *p < 0.05 vs. respective controls.

*DDR1 *mRNA is predicted to be a potential target of miR-199a-5p using PicTar (4-way), TargetScanS, and miRanda algorithm (Table 4) [24]. To assess whether miR-199a-5p can directly alter the expression of *DDR1 *luciferase reporter assays were employed. A fragment of the 3'-UTR of *DDR1 *mRNA, containing the putative miR-199a-5p-binding sequence, was cloned into a firefly luciferase reporter construct and co-transfected with a control β-gal reporter construct into HepG2 cells together with miR-199a-5p-specific precursor or control. A significant (p < 0.001) decrease in relative luciferase activity was observed when miR-199a-5p precursor was cotransfected with the luciferase reporter construct containing the fragment of the 3'-UTR of *DDR1 *mRNA. In contrast, no change in relative luciferase activity was observed in cells transfected with pMIR-REPORT™ firefly luciferase reporter control vector without the insert consisting of the 3'-UTR of *DDR1 *mRNA fragment (Figure 4A). These results indicate that miR-199a-5p can modulate gene expression directly via the *DDR1 *3'-UTR. In agreement with these data, qRT-PCR and western blotting shows that enhanced expression of miR-199a-5p as well as knockdown of *DDR1 *by siRNA result in a significant decrease of endogenous DDR1 mRNA and protein levels in HepG2 cells (Figures 4B and 5A). In mRNA stability assays we established that mir-199a-5p-mediated regulation of *DDR1 *in HepG2 cells is mainly achieved by degradation of *DDR1 *mRNA (Figure 5C). In contrast, transfection of miR-199a precursor in another hepatoma cell line, namely SNU-182, was not associated with an alteration of *DDR1 *mRNA expression (Figure 4C). However, a notable decrease of DDR1 protein levels became evident after miR-199a-5p precursor transfection (Figure 5B).

**Table 4:** *DDR1 *mRNA is predicted to be a potential target of miR-199a-5p by TargetScanS

	predicted consequential pairing of target region (top) and miRNA (bottom)
Position 1165-1185 of DDR1 3' UTR	5' ...AGAAAUAUAGGAUAGACACUGGA...
	| | | | | | |
hsa-miR-199a-5p	3' CUUGUCCAUCAGACUUGUGACCC
Position 1199-1219 of DDR1 3' UTR	5' ...GGAGCACCUGGGCCCCACUGGAC...
	| | | | | |
hsa-miR-199a-5p	3' CUUGUCCAUCAGACUUGUGACCC
Position 1260-1280 of DDR1 3' UTR	5' ...CUCUCUCCCUGUCACACACUGGA...
	| | | | | | |
hsa-miR-199a-5p	3' CUUGUCCAUCAGACUUGUGACCC
Position 1383-1403 of DDR1 3' UTR	5' ...CCUCCAUCACCUGAAACACUGGA...
	| | | | | | |
hsa-miR-199a-5p	3' CUUGUCCAUCAGACUUGUGACCC

### MiR-199a-5p differentially modulates invasion of hepatoma cell lines *in vitro*

**Figure 6:**
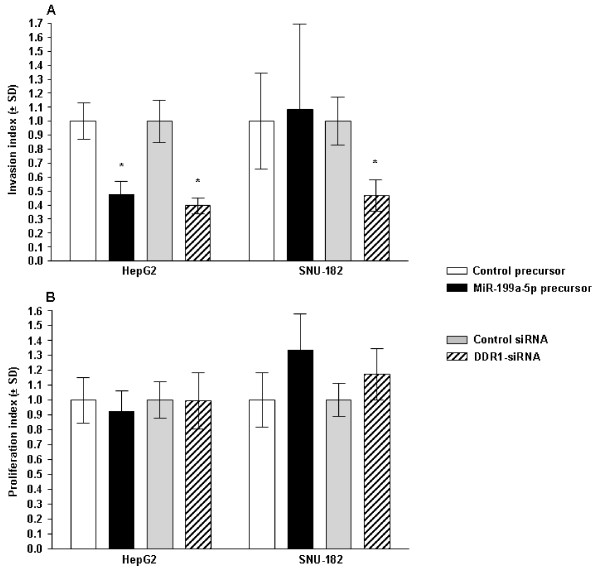
**Effects of miR-199a-5p and DDR1 expression on hepatoma cell invasion and proliferation**. **(A) **HepG2 and SNU-182 cells were transfected with 10 nM miR-199a-5p precursor or 100 nM *DDR1*-siRNA, and cell invasion across a membrane with 8 μm pores coated with Matrigel was assessed. Cells transfected with control miRNA precursor or control siRNA were used as controls. Data are expressed as invasion index and normalized to cell number using WST-1 assay. Data are shown from 3 separate experiments performed each in triplicate as mean ± SD. *p < 0.01 vs. respective controls. **(B) **HepG2 and SNU-182 cells were transfected with 10 nM miR-199a-5p precursor or 100 nM *DDR1*-siRNA, and cell and the proliferation was assessed after 72 hours using WST-1 assay. Cells transfected with control miRNA precursor or control siRNA were used as controls. Data are expressed as proliferation index. Data are shown from 3 separate experiments performed each in triplicate as mean ± SD.

We assessed the role of miR-199a-5p in tumor invasion by the ability of hepatoma cells to cross an extracellular matrix, a key determinant of malignant progression and metastasis formation. HepG2 cells were transfected with 10 nM miR-199a-5p precursor or 100 nM *DDR1*-siRNA, and invasive potential was assessed after 48 hours. Expression of miR-199a-5p precursors (p = 0.004) and *DDR1*-siRNA (p = 0.003) significantly decreased invasion of the HepG2 cell line (Figure 6A). These results suggest that altered expression of miR-199a-5p in HepG2 cells may contribute to increased cell invasiveness by functional deregulation of the activity of DDR1. However, only transfection of DDR1-siRNA (p = 0.002) but not of miR-199a-5p precursors (p = 0.820) significantly decreased invasion of another hepatoma cell line, namely SNU-182 (Figure 6A).

### MiR-199a-5p does not modulate proliferation of HepG2 and SNU-182 cells *in vitro*

To characterize the effect of miR-199a-5p on hepatoma proliferation, we performed overexpression studies using the miR-199a-5p specific precursor. Proliferation of HepG2 and SNU-182 cells was neither altered by precursor miR-199a-5p (p = 0.486 and p = 0.073, respectively) nor by *DDR1*-siRNA (p = 0.980 and p = 0.141, respectively) (Figure 6B). These studies indicate that DDR1 is rather involved in the process of tumor cell invasion than in tumor growth.

## Discussion

Decreased miR-199a-5p expression in HCC has been repeatedly reported, but its functional relevance has not been elucidated to date [11,12,15]. A pivotal role for miRNAs in the process of malignant transformation has been suggested in the literature [9,31]. However, the precise molecular mechanisms by which miRNAs modulate tumor cell biology are largely unknown. MiRNAs from animals were first reported to repress translation without affecting mRNA levels [32]. More recent evidence indicated that miRNAs and siRNAs can control post-transcriptional gene expression by directing the endonuclease cleavage of target mRNA, which is referred to as "slicer" activity [33]. Endonucleolytic cleavage is generally favored by perfect base-pairing between miRNA and mRNA. Some mismatches, however, can be tolerated and still allow endonucleolytic cleavage to occur [34]. The majority of animal miRNAs are only partially complementary to their targets [35]. Several reports have shown that animal miRNAs can also induce significant degradation of target mRNAs despite imperfect mRNA-miRNA base-pairing [36,37], referred to as "slicer"-independent decay. This phenomenon emphasizes mRNA degradation as an important aspect of miRNA-mediated repression of gene expression. There is also some evidence that "slicer"-independent mRNA decay induced by miRNAs might occur through promotion of mRNA decapping and 5'-3'-degradation [33]. The contribution of translational repression or mRNA degradation to gene silencing appears to differ for each miRNA:target pair and is likely to depend on the particular set of proteins bound to the 3'UTR of the mRNA [38]. MiR-199a-5p, which is partially complementary to the 3' UTR of *DDR1 *mRNA, induced significant degradation of *DDR1 *mRNA in hepatoma cells in our study. Thus, *DDR1 *has been experimentally validated as a target gene of miR-199a-5p. MiR-199b-5p, which has a very similar nucleotide sequence, is also predicted to target *DDR1*. However, miR-199b-5p was only detectable in four out of 24 tissue samples in a miRNA microarray assay (data not shown) and it has never been reported to be de-regulated in HCC in literature. Therefore, the regulatory function of miR-199b-5p was not further assessed in this study.

DDR1 is a tyrosine kinase receptor for collagen [22] and its activation can cause tumor invasion which appears to be mediated by matrix metalloproteinases (MMP)2 and MMP9 [19,23]. Indeed, we found a positive correlation between the expression of *DDR1 *and *MMP2 *in our patient cohort (data not shown), further supporting the important role of DDR1 for tumor invasion. Consistent with recently published findings [23] and our results indicating a critical role for DDR1 in HCC progression, we found a significant correlation between the expression of *DDR1 *and AJCC/UICC tumor stage. Analyzing predictive factors for advanced tumor stage at the time of HCC diagnosis, we found a positive correlation between AJCC/UICC stage III-IV and poor tumor differentiation, presence of macrovascular invasion, and high *DDR1 *expression. In addition, multivariate logistic regression analysis identified high *DDR1 *expression as the single independent factor associated with advanced tumor stage, and, hence, poor prognosis. In line with this clinicopathological observation, we found that *DDR1 *gene silencing by transfection of miR-199a-5p into HepG2 cells significantly decreased tumor cell invasion *in vitro*. However, our studies also indicate that DDR1 is rather involved in the process of tumor invasion than in tumor growth. These results demonstrated that miR-199a-5p is capable to modulate tumor cell invasion at least in part by targeting *DDR1*. Although in our study *DDR1 *has been validated as a target gene of miR-199a-5p, no significant correlation between miR-199a-5p and *DDR1 *mRNA expression was found in tumor samples from our patient cohort. In addition, SNU-182 hepatoma cells exhibited increased levels of expression of both miR-199a-5p and *DDR1 *mRNA. Transfection of miR-199a-5p did not induce a change in *DDR1 *mRNA expression, but significantly down-regulated DDR1 protein in SNU-182 cells. However, no significant inhibitory effect on tumor invasion was noted. Considering the preexisting high expression of miR-199a-5p in SNU-182 cells, our results might hint at a certain independence of *DDR1 *to miR-199a-5p-mediated gene regulation and function in these cells. Our data hint at a more complex regulation network between *DDR1 *and miR-199a-5p in HCC. One reason might be that HCC is a very heterogeneous tumor entity and distinct cellular components might interfere with the effect of miR-199a-5p on DDR1 [39]. For instance, by the use of miRNA target prediction algorithms other 23 miRNAs were also predicted to target DDR1 [24]. In addition, DDR1 has been demonstrated to be a direct transcriptional target of the p53 tumor suppressor gene [40] and, therefore, the p53 status in tumor cells may also affect the expression of *DDR1*. Finally, recent findings reveal a more diverse role for small RNA molecules in the regulation of gene expression than previously recognized. For instance, miRNAs can act also as translation activators under specific cellular conditions [41]. In addition, double strand RNAs can activate rather than repress gene expression by targeting non-coding regulatory regions in gene promoters [42]. To date, the only reported stimulatory effect of miRNA on RNA expression is represented by the interaction between miR-122 and replication of hepatitis C virus RNA in hepatocytes [43]. MiR-199a-5p expression has also been shown to be diversely deregulated in other cancer types. For instance, miR-199a-5p was also found to be down-regulated in ovarian cancer [44] and oral squamous cell carcinoma [45], but up-regulated in cervical carcinoma [46] and bronchial squamous cell carcinoma [47]. Moreover, increased expression of miR-199a-5p has been considered a signature for high metastatic risk and a poor prognosis in uveal melanoma [48].

Thus, the complexity of the regulation of mRNA by miRNA encountered in our and other studies indicates that the effect of miRNA on its target gene is cell type and environment dependent. However, our study demonstrates a previously uncharacterized biological function of miR-199a-5p such as the ability to inhibit tumor invasion through targeting *DDR1*.

Less than half of patients with HCC are eligible for potential curative treatment including liver resection and transplantation because of advanced tumor stage at time of diagnosis. The combination of clinical and biological predictors may increase diagnostic accuracy of tumor staging, thus permitting optimized therapeutic management of HCC patients. Although *DDR1 *expression was shown to be the only predictive factor for advanced HCC, our study was clearly limited by the small sample size which may tend to overestimate the prognostic value of *DDR1 *expression. Thus, prospective studies that seek and independently validate the prognostic utility of *DDR1 *expression for patients with HCC in a larger and carefully selected cohort should be conducted. Patient survival after surgical treatment is hampered by frequent tumor recurrence and systemic chemotherapy is largely ineffective [49]. In recent years, kinase inhibitors have become an attractive target class for drug development [50], and it was shown recently that systemic application of a multikinase inhibitor improves survival of patients with HCC [51]. Further investigation of therapeutic strategies targeting the miR-199a-5p-*DDR1 *signaling network is therefore warranted. In conclusion, identification of the miR-199a-5p:*DDR1 *target pair and its crucial role in tumor cell invasion highlight the translational relevance for both prognostic prediction and targeted molecular therapy for patients with HCC.

## Abbreviations

AJCC/UICC: American Joint Committee on Cancer and International Union Against Cancer; AUROC: area under receiver operating characteristics; β-gal: beta-galactosidase; CI: confidence interval; DDR1: discoidin domain receptor-1; GAPDH: glyceraldehyde-3-phosphate dehydrogenase; HCC: hepatocellular carcinoma; HMBS: hydroxymethyl-bilane synthase; HPRT1: hypoxanthine phosphoribosyl-transferase 1; MGB: minor grove binder; miRNA: micro-ribonucleic acid; miR-199a-5p: microRNA excised from the 5' arm of microRNA-199a precursor; NT: non-tumor tissue; qRT-PCR: quantitative real-time reverse transcription polymerase chain reaction; RIN: RNA integrity number; *RNU44*: small nucleolar RNA C/D box 44; SD: standard deviation; SDHA: succinate dehydrogenase complex, subunit A; SE: standard error; siRNA: short interfering RNA; UTR: untranslated region; WST: water soluble tetrazolium.

## Competing interests

The authors declare that they have no competing interests.

## Authors' contributions

QS, VRC and SB designed, performed, and analyzed the research and wrote the paper. XZ and ML performed additional substantial experiments and critically reviewed the manuscript. SI performed statistical analysis. FW, GCS and AR participated in patient sample collection and data analysis; GG and AP conceived the study, participated in its design and helped to draft the manuscript. All authors read and approved the final manuscript.
